# Dr. Frederick Tiedemann

**Published:** 1861-05

**Authors:** 


DR. FREDERICK TIEDEMANN, formerly Professor of Anatomy and Physiology in the University of Heidelberg, died on the 22d of January, at Munich, in the eightieth year of his age. He was one of the most distinguished medical men of his day in Germany. He was born in August, 1781, at Kassel, took his medical degree at Marburg, in 1804, and in 1805 was appointed Professor of Anatomy and Zoology in the University of Landshut. In 1816 he was called to Heidelberg as Professor of Zoology, Anatomy, and Physiology, and it was here that he earned his world-wide reputation as a teacher and an author. In 1818 he was honored with an invitation to the University of Bonn, then recently founded; and in 1833 a similar distinction was conferred upon him by the University of Berlin, on the death of Rudolphi. The place thus declined was subsequently bestowed upon Johannes Müller, the great physiologist. In 1849 Tiedemann was overwhelmed with grief by the death of a son, who, in consequence of having taken a part in the revolution of that period, was shot by order of the Grand-ducal Government, which the father had so faithfully served for the third of a century. Soon after this sad event he retired to Frankfort-on-the-Main, where he remained until 1856, having two years previously celebrated his doctor's jubilee, a great festival, given him by the physicians of that and the neighboring cities. The University of Marburg also, in compliance with a time-honored German custom, renewed his medical diploma. He now went with his son-in-law, Professor Bischoff, to Munich, where, as already stated, he closed his long and useful career, early in the present year, the immediate cause of his death being pneumonia. Tiedemann was a prolific author. Quite a number of his writings have been translated into foreign languages. His latest publication was a small treatise on the use and abuse of tobacco. His great work on the anatomy of the arteries, issued in 1822, is in every public medical library. Its illustrations are unrivaled. He was a man of the purest character, of vast industry and perseverance, a great anatomist and physiologist, and an eminently successful teacher. We subjoin, from Dr. Engelmann's Bibliotheca Medico-Chirurgica et Anatomico-Physiologica, issued in 1848, and a copy of which is in our library, a list of Professor Tiedemann's works, in order to afford our readers a better idea of forming a correct estimate of his immense labors.

Anatomie der kopflosen Missgeburten. Mit 4 Kpftaf. Fol. *Landshut* 1813.

Anatomie u. Bildungsgeschichte des Gehirns im Fötus des Menschen, nebst ein. vergleichenden Darstel. des Hirnbaues in den Thieren. Mit 7 Kpftaf. gez. v. Dr. *Mänz* gr. 4. *Nürnburg* 1816. Physiologie des Menschen. 1. u. 3. Bd gr. 8. *Darmstadt* 1830, 36.

1. Bd. Allgem. Betrachtgn. der organ. Körper. 1830.

3. Bd. Untersuchungen üb. das Nahrungs-Bedürfniss, den Nahrungs-Trieb u. die Nahrungs-Mittel des Menschen. 1836.

Der 2. Bd. ist noch nicht erschienen.

Abbildungen üb. den Verlauf der Pulsadern des menschl. Körpers, sammt erklär. Texte (in 4.) in lat. u. deutscher Sprache.—A. u. d. Tit.: Explicationes tabular. arteriarum corporis humani. Nach d. Natur in Lebensgrösse, gez. v. *Roux* u. lithogr. 4 Lieff. Mit 78 (wovon 38 illum.) Taf. in Imp.-Fol. *Carlsruhe* 1822—24.

Neue (Tit.-) Ausg. in 12 Lieff. 1842.

Explicationes supplementorum ad tabulas arteriarum corporis humani.—A. u. d. Tit.: Erklärungen der Ergänzungen zu den Abbildungen der Pulsadern des menschl. Körpers. (15 lith. Taf,: XXXIX—LIII. in gr. fol. u. 17¼ Bog. deutsch. u. lat. Text in 4.) *Heidelberg* 1846.

Beobachtungen üb. die hohe Theilung der Armschlagader in die Speichen u. Ellenbogen-Schlagader. gr. 4. *München* 1820.

Tabulæ nervorum uteri. Cum IV tabb. aen. Fol. maj. *Heidelberg.* 1823.

Das Hirn des Negers mit dem des Europäers u. Orang-Outangs verglichen. Mit 6 (lith.) Taf. Imp.-4. *Heidelberg* 1837.

Von den Duverneyschen, Bartholinschen od. Cowperschen Drüsen des Weibes u. der schiefen Gestaltung u. Lage der Gebärmutter. Mit 4 (5) Taf. Abbildgn. gr. 4. *Heidelberg* 1840.

Von der Verengung u. Schliessung der Pulsadern in Krankheiten. Mit 3 (lith.) Taf. (in gr. Fol.) Imp.-4. *Ebend.* 1843.

Von Lebenden Würmern u. Insekten in den Geruchs Organen des Menschen, den Zufällen, welche sie verursachen, u. den Mitteln sie auszutreiben. (Vorgetragen am 24. Mai 1844 in der Gesellschaft f. Natur u. Heilkunde zu Heidelberg.) gr. 8. *Mannheim* 1844.

u. L. Gmelin, Versuche üb. die Wege, auf welchen Substanzen aus dem Magen u. Darmkanal ins Blut gelangen, üb. die Verrichtungen der Milz u. die geheimen Harnwege. gr. 8. *Heidelberg* 1820.

u. L. Gmelin, Die Verdauung, nach Versuchen. 2 Bde. 2. wohlf. (Tit.-) Ausg. gr. 4. *Heidelberg* (1826, 27.) 1831.

s. auch: Bericht der Naturforscher in Heidelberg.—Zeitschrift f. Physiologie.

